# Pangenome-Wide Identification, Evolutionary Analysis of Maize ZmPLD Gene Family, and Functional Validation of ZmPLD15 in Cold Stress Tolerance

**DOI:** 10.3390/plants14243858

**Published:** 2025-12-18

**Authors:** Si-Nan Li, Yun-Long Li, Ming-Hao Sun, Yan Sun, Xin Li, Quan Cai, Yunpeng Wang, Jian-Guo Zhang

**Affiliations:** 1 Maize Research Institute, Heilongjiang Academy of Agricultural Sciences, Harbin 150086, China; 2 Institute of Agricultural Biotechnology, Jilin Academy of Agricultural Sciences (Northeast Agricultural Research Center of China), Changchun 130033, China

**Keywords:** *Zea mays*, PLD, ZmPLD15, pangenome, cold tolerance, photosynthetic system protection, SV

## Abstract

*Phospholipase D (PLD)* genes play key roles in plant abiotic stress responses, but the systematic identification of the maize (*Zea mays*) PLD family and its cold tolerance mechanism remain unclear. Using 26 maize genomes (pangenome), we identified 21 ZmPLD members via Hidden Markov Model (HMM) search (Pfam domain PF00614), including five private genes—avoiding gene omission from single reference genomes. Phylogenetic analysis showed ZmPLD conservation with Arabidopsis and rice PLDs; Ka/Ks analysis revealed most *ZmPLDs* under purifying selection, while three genes (including *ZmPLD15*) had positive selection signals, suggesting roles in maize adaptive domestication. For *ZmPLD15*, five shared structural variations (SVs) were found in its promoter; some contained ERF/bHLH binding sites, and SVs in Region1/5 significantly regulated *ZmPLD15* expression. Protein structure prediction and molecular docking showed conserved ZmPLD15 structure and substrate (1,2-diacyl-sn-glycero-3-phosphocholine) binding energy across germplasms. Transgenic maize (B73 background) overexpressing ZmPLD15 was generated. Cold stress (8–10 °C, 6 h) and recovery (24 h) on three-leaf seedlings showed transgenic plants had better leaf cell integrity than wild type (WT). Transgenic plants retained 45.8% net photosynthetic rate (Pn), 47.9% stomatal conductance (Gs), and 55.8% transpiration rate (Tr) versus 7.6%, 21.3%, 13.8% in WT; intercellular CO_2_ concentration (Ci) was maintained properly. This confirms ZmPLD15 enhances maize cold tolerance by protecting photosynthetic systems, providing a framework for *ZmPLD* research and a key gene for cold-tolerant maize breeding.

## 1. Introduction

The phospholipase gene family serves as a core regulatory hub in plant metabolism and signal transduction. By hydrolyzing phospholipids, it generates a range of bioactive molecules, including free fatty acids (FFAs) [1], phosphatidic acids (PAs) [2], diacylglycerol (DAG) [3], and lysophospholipids [4], which collectively modulate multiple processes such as plant growth and development [1], stress responses [5,6], and lipid metabolism [7]. Based on their distinct hydrolysis sites, phospholipases are categorized into three major classes: phospholipase A (PLA) [8], phospholipase C (PLC) [9], and PLD [10]. Among these, PLD stands out for its role as a “signal hub” in plant responses to various abiotic and biotic stresses, acting as a key node linking stress perception, signal transduction, and resistance expression, thus attracting significant research attention [2,6,10]. Specifically, PLD cleaves phosphatidylcholine (PC) to produce PA and choline: PA functions as a central signaling molecule regulating membrane dynamics and energy metabolism, while choline contributes to the synthesis of osmoprotectants [11]. Additionally, PLD directly maintains membrane structure [12], enabling it to both sustain basic cellular functions by preserving membrane stability and activate downstream stress response pathways via the production of key signaling molecules [13].

To date, genome-wide identification of *PLD* genes has been successfully accomplished in a variety of crop species. For instance, 12 *PLD* genes have been identified in *Arabidopsis thaliana* (Arabidopsis) [14], 17 in *Oryza sativa* (rice) [15], 18 in *Glycine max* (soybean) [16], and 22 in *Zea mays* (maize) [17]. In the model plants Arabidopsis and rice, the PLD gene family is classified into seven isoforms, namely “α”, “β”, “γ”, “δ”, “φ”, “ζ”, and “κ”, based on their physicochemical properties and sequence structure characteristics [10,18]. Structurally, PLD proteins possess two key domains: a C-terminal catalytic domain containing two HKD motifs, which is essential for their hydrolytic activity, and an N-terminal domain that can be either a Phox homology/Pleckstrin homology (PX/PH) domain or a C2 domain [2]. According to the type of N-terminal domain, PLD proteins are further divided into two subfamilies: the C2-PLD subfamily and the PX/PH-PLD subfamily [10,18]. Notably, the majority of identified PLD proteins rely on the binding of calcium ions (Ca^2+^) and phospholipids to activate their hydrolytic activity, highlighting the significance of these cofactors in regulating PLD function [18]. This structural and functional classification provides a solid foundation for understanding the diverse roles of *PLD* genes across different plant species.

Beyond their structural and catalytic features, *PLD* genes and their hydrolytic product, PA, have been demonstrated to interact with a suite of key genes involved in plant phospholipid signaling pathways. These interacting partners include the heterotrimeric G protein Gα subunit (Gα), the aspartic protease Cardosin A, NADPH oxidase, and sphingosine kinase (SPHK). Such interactions underscore the central role of PLD in integrating multiple signaling cascades [2,19]. As one of the most crucial “second messengers” in plant stress resistance, PA exhibits the characteristics of “multi-target regulation and strong regulatory capacity”. It can directly bind to and activate core signaling pathway kinases, such as mitogen-activated protein kinases (MAPKs) and calcium-dependent protein kinases (CDPKs), thereby initiating downstream stress defense responses [20,21]. Additionally, PA plays a pivotal role in regulating ion channels. For example, it can modulate K^+^/Na^+^ transporters (e.g., SOS1) located on the cell membrane, aiding plants in extruding excess Na^+^ under salt stress and maintaining cellular ion homeostasis [22]. Simultaneously, PA can regulate Ca^2+^ channels, triggering the “calcium signal”—an early core signal for plants to perceive environmental stresses [23]. These multifaceted regulatory roles of PA, mediated by PLD, make the PLD-PA signaling module a critical player in plant adaptation to adverse environmental conditions [13]. Extensive experimental studies have been conducted to characterize the stress resistance functions of the *PLD* gene family in the model plant *Arabidopsis thaliana*, yielding valuable insights. For example, members of the *PLDα* and *PLDδ* isoforms have been shown to be involved in responses to abiotic stresses, such as salt stress and drought stress [6,10,24]. These isoforms contribute to enhancing plant tolerance by regulating processes like membrane stability and stress signal transduction [25]. Furthermore, *PLDα1* and *PLDδ* are implicated in key physiological processes, including stomatal closure, cell senescence, and cell death [6]. Stomatal closure, in particular, is a vital mechanism for plants to reduce water loss under drought conditions, emphasizing the practical importance of these PLD isoforms in plant water use efficiency [26]. In terms of nutrient stress adaptation, PLDε has been found to promote the elongation of primary roots and root hairs under low-nitrogen conditions, facilitating plants’ ability to absorb nutrients from the soil [27]. On the biotic stress front, PLDβ is associated with the defense response against fungal pathogen infection, highlighting the role of *PLD* genes in plant-pathogen interactions [28]. These comprehensive studies on Arabidopsis *PLD* genes not only deepen our understanding of the molecular mechanisms underlying plant stress resistance [29] but also inspire further exploration into the identification and functional characterization of *PLD* gene families in various crop species [30]. Moreover, the findings provide essential experimental data for the development of genetically engineered crops with enhanced stress tolerance, which is of great significance for improving crop yield and quality under changing environmental conditions [31].

As a globally crucial crop, maize is severely affected by cold stress, which impairs its yield and limits its planting range [32]. Thus, identifying cold stress-related gene families, especially the phospholipase gene family, is of great significance. By specifically hydrolyzing phospholipids to produce key signaling molecules—including FFAs, IP_3_ (which triggers Ca^2+^ signaling), and PA—PLD modulates a multi-tiered cold tolerance network that spans from cellular protection (e.g., membrane stability maintenance) to developmental adaptation (e.g., reproductive stage energy allocation) [10]. This network coordinately maintains membrane lipid homeostasis (e.g., ZmPLA1-3 stabilizes thylakoid membranes, ZmPLDα preserves the cytoskeleton) [33], modulates hormone signaling (e.g., PLA provides precursors for JA synthesis) [34], mediates calcium signal transduction (e.g., PLC activates the CDPK pathway) [35], and regulates energy allocation during the reproductive stage (e.g., ZmpPLA-7 improves seed setting rate under cold stress) [36,37]. Studying this network not only reveals the “lipid–signal–metabolism” synergy mechanism underlying maize cold resistance but also provides multiple targets for molecular breeding (e.g., *ZmPLA1-3* promoter markers, *PLD* gene editing) [38,39], facilitating the development of cold-tolerant varieties to address climate change challenges [40].

Only a few studies have reported the identification and analysis of the maize *PLD* gene family based on a single reference genome, and this approach fails to detect family members absent from the reference genome but present in other genomes. Hufford et al. constructed a maize pangenome using 26 high-quality genomes, which contains abundant presence–absence variations (PAVs) and SVs, laying a foundation for studying the *PLD* gene family and its functions in the maize pangenome [41]. In this study, we successfully identified 21 *PLD* pan-genes from the pangenome of 26 high-quality maize genomes, including nine core genes, two near-core genes, five dispensable genes, and five private genes (lineage-specific genes present in only one of the 26 genomes). We analyzed the non-synonymous substitution rate (Ka), synonymous substitution rate (Ks), and their ratio (Ka/Ks) of PLD family members across 26 varieties, the transcription factor (TF) interaction in the promoter SV region, and protein structure prediction combined with molecular dynamics, investigating the effects of SVs on promoter and gene structure as well as expression. Additionally, through a cold stress model, we analyzed the co-expressed TFs of *PLD* genes, providing theoretical support and data reference for subsequent functional identification of the *PLD* family in the pangenome.

## 2. Results

### 2.1. Pan-Genome-Wide ZmPLD Identification

To systematically characterize the *PLD* pan-gene family in maize, a comprehensive identification was conducted using 26 high-quality reference genomes. A total of 21 *PLD* pan-genes were successfully identified, and these genes were further classified into four categories based on their presence frequency across the 26 genomes: nine core genes (present in all 26 genomes), two near-core genes (present in most genomes with a high frequency), five dispensable genes (present in a subset of genomes), and five private genes (present in only one or a few specific genomes). Detailed information regarding the classification, gene IDs, and corresponding genome sources of these 21 *PLD* pan-genes is provided in Supplementary Table S1, which serves as a fundamental dataset for subsequent functional and evolutionary analyses.

**Figure 1:**
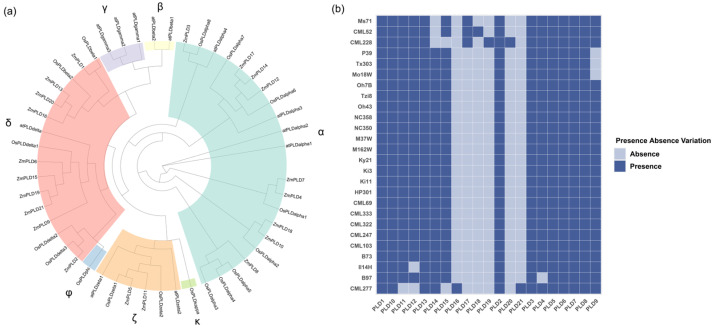
Phylogenetic analysis and PAVs of *PLD* genes in maize, rice, and Arabidopsis. (**a**) Phylogenetic tree of *PLD* genes from maize, rice, and Arabidopsis. Different colors indicate that the corresponding clades belong to the “α”, “β”, “γ”, “δ”, “φ”, “ζ”, and “κ” groups. (**b**) Heatmap showing the presence and absence of *ZmPLD* genes across 26 maize varieties. Dark blue indicates that the corresponding *ZmPLD* gene member is present in the genome, while light blue indicates absence.

To explore the evolutionary relationships of *ZmPLD* gene family members, a phylogenetic tree was constructed by aligning the protein sequences of the 21 maize *PLD* pan-genes with previously reported PLD protein sequences from *Arabidopsis thaliana* (a model dicot plant) and *Oryza sativa* (rice, a model monocot plant). This cross-species phylogenetic analysis aimed to clarify the evolutionary divergence and subtype classification of maize *PLD* genes. As shown in Figure 1a, the maize PLD proteins were clustered into three distinct PLD subtypes, reflecting their evolutionary conservation and functional differentiation. Specifically, nine of the 21 *ZmPLD* genes belonged to the α subtype, 10 genes were classified into the δ subtype, and the remaining 2 genes were grouped into the ζ subtype. This subtype distribution pattern provides insights into the functional specialization of *ZmPLD* genes, as different PLD subtypes are typically associated with distinct physiological roles in plants.

In addition to evolutionary analysis, the PAVs of *ZmPLD* genes across the 26 maize varieties was investigated to assess the genetic diversity of the *PLD* gene family. Figure 1b illustrates the PAV profile of each *ZmPLD* gene in the 26 genomes. Among the 21 *ZmPLD* pan-genes, several exhibited strict private gene characteristics: *PLD16* was exclusively present in the CML277 genome, PLD18 was unique to CML52, PLD19 and PLD20 were only detected in CML228, and PLD21 was specific to CML277. Notably, PLD17 showed a slightly broader distribution, being present in three genomes, namely CML52, CML228, and Ms71. This PAV pattern not only highlights the significant genetic variation in the *PLD* gene family among different maize varieties but also implies that certain *ZmPLD* genes may be associated with specific adaptive traits of their host varieties.

### 2.2. ZmPLD Is Subjected to Different Selection Pressures Among Maize Varieties

**Figure 2:**
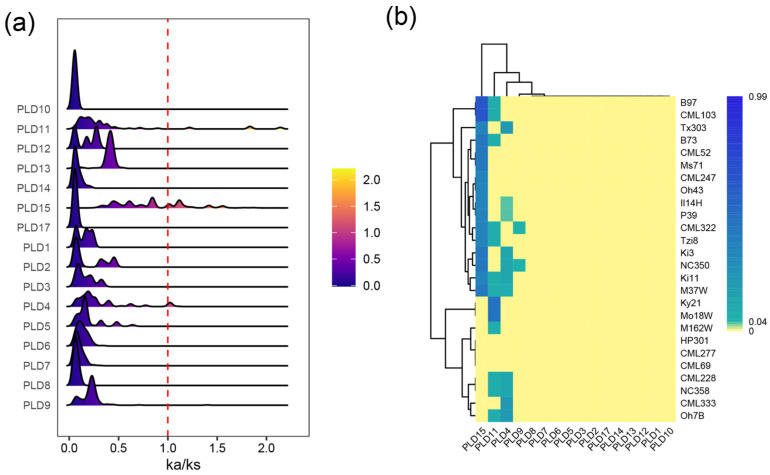
Ka/Ks values of *ZmPLD* genes. (**a**) Distribution of Ka/Ks values of *ZmPLD* genes in 26 maize varieties, but 5 private genes were excluded because each belongs to a subfamily with only one member (Ka/Ks calculation requires ≥2 members per subfamily for reliable pairwise sequence comparison). Detailed information on private genes is provided in Supplementary Table S1. The vertical dashed line indicates the threshold value of Ka/Ks = 1.0, which is used to distinguish the evolutionary selection pressure types of different ZmPLD pan-gene family members. Values to the right of the line suggest positive selection, and values to the left indicate purifying selection. (**b**) Heatmap showing the occurrence frequency of Ka/Ks > 1 for each *PLD* gene among different maize varieties.

During the long-term breeding process of maize, distinct cultivation conditions (such as ecological environments, agronomic management, and target traits) across different varieties have led to variations in the selection pressure acting on functional genes. The *ZmPLD* gene family, as a key player in regulating plant stress responses and growth development, is inevitably affected by such selection pressure, which may further drive the functional differentiation of its members among varieties. To explore the selection pressure on *ZmPLD* genes during maize cultivar breeding and clarify their evolutionary trends, we first screened *ZmPLD* gene family members that existed in at least two maize varieties (to ensure reliable sequence alignment). Then, we performed pairwise sequence alignment of the same gene family members across different varieties and calculated the Ka/Ks for each *ZmPLD* gene. The Ka/Ks ratio is a classic indicator to evaluate evolutionary pressure: a ratio greater than 1 indicates positive selection (advantageous mutations are retained), a ratio equal to 1 suggests neutral evolution (mutations are not affected by selection), and a ratio less than 1 reflects purifying selection (deleterious mutations are eliminated). Detailed results of this analysis are presented in Figure 2.

The distribution of Ka/Ks values of *ZmPLD* genes across the 26 maize cultivars is shown in Figure 2a. Among the 21 identified *ZmPLD* pan-genes, five were private genes that existed in only one variety; due to the lack of homologous sequences in other varieties, their Ka/Ks values could not be calculated. For the remaining 16 genes, four genes (*ZmPLD4*, *ZmPLD9*, *ZmPLD11*, and *ZmPLD15*) exhibited a high proportion of Ka/Ks values greater than 1. This result indicates that these four *ZmPLD* genes were under positive selection during maize domestication and cultivar breeding. Positive selection usually implies that the mutations of these genes may bring adaptive advantages to maize (e.g., enhanced stress resistance or improved agronomic traits), leading to the active retention of these variant alleles in the population and thus showing distinct variation trends. In contrast, the Ka/Ks values of all other *ZmPLD* genes were in the range of 0–1, which is a typical signature of purifying selection. Purifying selection suggests that these *ZmPLD* genes play essential and conserved roles in maize growth and development (e.g., maintaining basic membrane lipid metabolism or signal transduction), and any deleterious mutations in these genes would be eliminated by natural or artificial selection to ensure the stability of their core functions during cultivar breeding.

### 2.3. Expression and Structure of ZmPLD15 Genes Are Affected by SV in the Promoters

**Figure 3:**
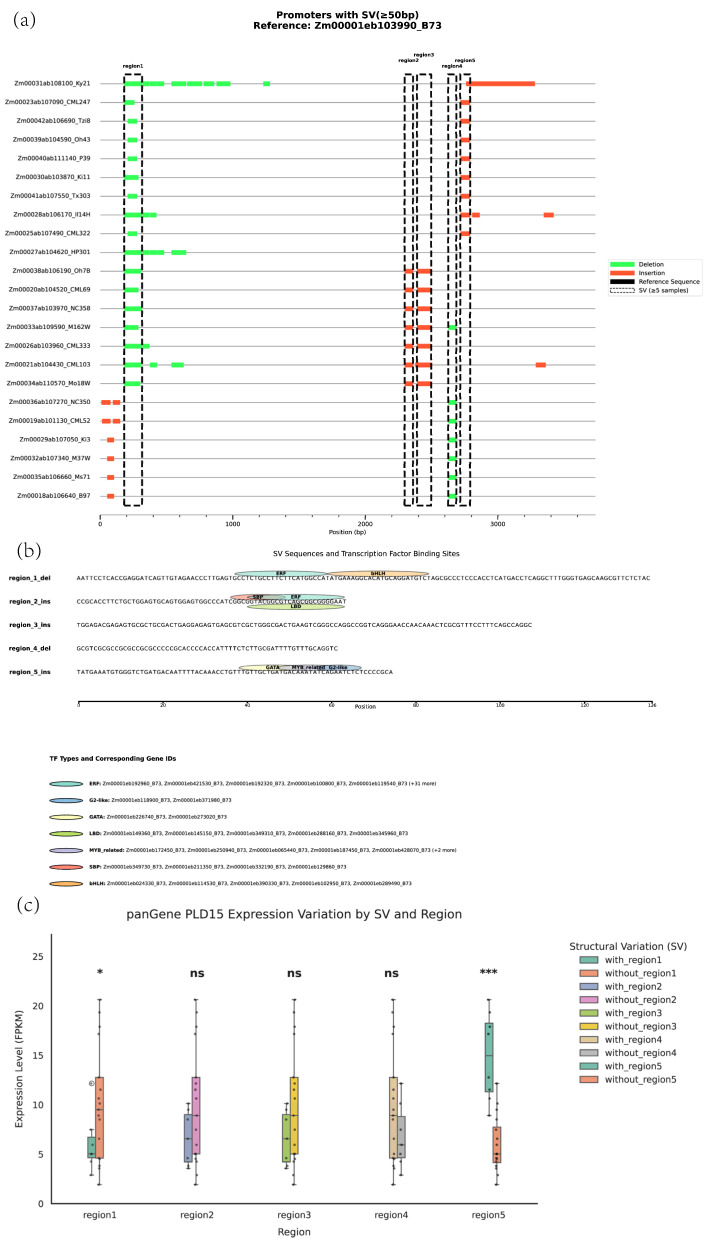
SV analysis of the *PLD15* gene promoter and its effects on gene expression. (**a**) SV analysis in the promoter region of the *PLD15* gene. (**b**) Analysis of TF binding sites in the SV sequences of the *PLD15* promoter. (**c**) Analysis of the effect of the presence/absence of different SV sequences on the expression of the *PLD15* gene. Dots represent the PLD15 gene expression levels of individual samples in each SV presence/absence group, and lines represent the mean ± standard deviation (mean ± SD) of the expression levels in each group. Statistical significance was determined by Student’s *t*-test (* *p* < 0.05, *** *p* < 0.001, ns = no significant difference). Among the five SV regions: region1 significantly affects the expression of PLD15 gene (*), region5 extremely significantly affects the expression of PLD15 gene (***), and region2, region3 and region4 have no significant effect on the expression of PLD15 gene (ns).

To investigate the potential impact of promoter SVs on *ZmPLD15* expression, we focused on the 2000 bp upstream region of *ZmPLD15* (a key candidate gene with positive selection signals) and compared it across 23 maize genomes relative to the reference genome B73 in Figure 3a. A total of five shared SVs were identified, with the criteria of being present in at least 5 maize accessions and involving insertions/deletions (Indels) longer than 50 bp. These shared SVs were further categorized based on their genetic characteristics: Region1 and Region4 were classified as deletion-type SVs (i.e., specific DNA segments missing compared to the B73 promoter), while Region2, Region3, and Region5 were defined as insertion-type SVs (i.e., additional DNA segments present relative to the B73 promoter). This classification provides a foundation for subsequent analyses of how different SV types regulate *ZmPLD15* transcription.

To decipher the regulatory mechanisms underlying these promoter SVs, we employed FIMO (Find Individual Motif Occurrences) analysis to predict TF binding sites within each SV region. The results, visualized in Figure 3b, revealed distinct TF binding profiles across the SV regions. Specifically, Region1 was predicted to harbor binding sites for ERF (Ethylene Response Factor) and bHLH (basic Helix–Loop–Helix) TFs, which are well-known regulators of plant stress responses and growth development. Region2 showed potential binding sites for ERF, LBD (Lateral Organ Boundaries Domain), and SBP (SQUAMOSA Promoter Binding Protein) TFs, with LBD and SBP TFs playing critical roles in plant morphogenesis and signal transduction. Region5 was found to contain putative binding motifs for GABA (Gamma-Aminobutyric Acid)-responsive TFs, G2-like TFs, and MYB-related TFs, which are involved in abiotic stress adaptation and secondary metabolism. In contrast, no TF binding sites were detected in Region3 and Region4, suggesting that these two SV regions may not directly mediate transcriptional regulation through TF interactions.

To further validate whether these SV regions affect *ZmPLD15* expression, we classified 24 maize cultivars into different groups based on the presence or absence of each SV-related sequence. We then performed a significance analysis of *ZmPLD15* expression levels between groups with and without the SV sequences. As shown in Figure 3c, only the SVs in Region1 and Region5 significantly influenced *ZmPLD15* expression levels. Specifically, cultivars carrying the Region1 deletion or Region5 insertion exhibited significantly different *ZmPLD15* expression patterns compared to those without these SVs. This result indicates that Region1 and Region5 are functional SV regions that modulate *ZmPLD15* transcription, likely through their interactions with specific TFs (as predicted in the FIMO analysis). In contrast, the presence or absence of SVs in Region2, Region3, and Region4 did not lead to significant changes in *ZmPLD15* expression, further supporting that not all promoter SVs contribute to transcriptional regulation—only those with functional TF binding sites may play a role in gene expression control.

### 2.4. Impact of Mutations on ZmPLD15 Protein Spatial Structure and Substrate Binding Free Energy

**Figure 4:**
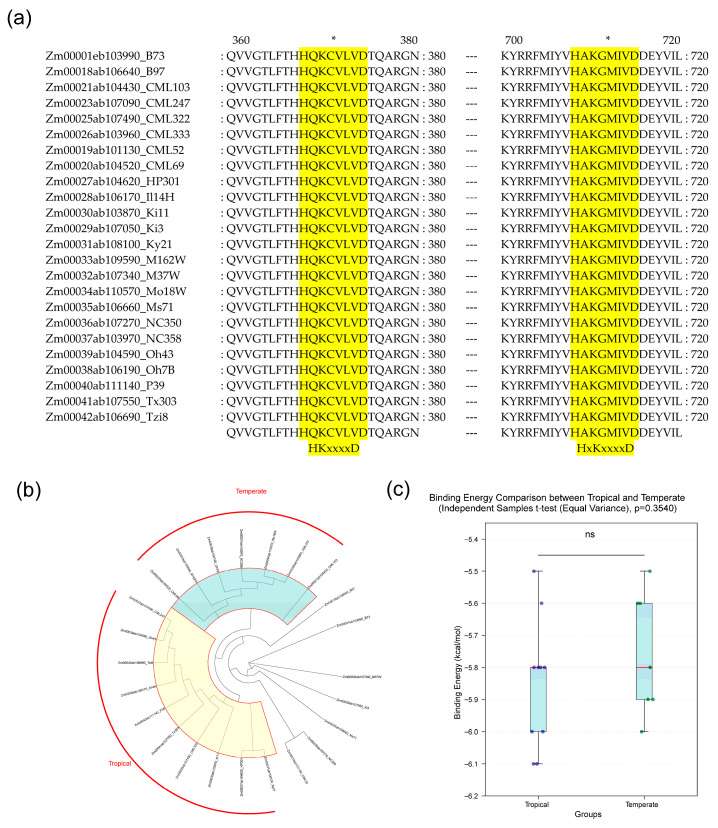
Structural simulation, molecular docking, and evolutionary analysis of ZmPLD15 proteins from different maize genomes. (**a**) sequence alignment of ZmPLD15 proteins derived from different maize genomes containing conserved HKD motifs. Asterisks (*) indicate the locations of HKD motifs sequences, which are key functional domains responsible for the phospholipase D activity of ZmPLD proteins. (**b**) Phylogenetic tree of ZmPLD15 proteins from different maize genomes. Teal indicates that all ZmPLD15 pan-gene family members in the corresponding clade are derived from temperate regions, while yellow indicates that all members in the clade are derived from tropical regions. (**c**) Analysis of significant differences in substrate binding free energy of ZmPLD15 proteins from tropical and temperate maize germplasms. Dots represent the statistical analysis results of substrate binding affinity of ZmPLD15 proteins among maize germplasms from different accumulated temperature zones, and lines represent the mean ± SD. Statistical significance was determined by Student’s *t*-test (*p* < 0.05, ns = no significant difference), and no significant difference was observed between the two groups.

To explore whether SVs in the *ZmPLD15* gene affect its protein structure (primary to tertiary) and subsequent substrate binding ability, we first predicted the sequence conservation and the three-dimensional (3D) structures of ZmPLD15 proteins encoded by 24 different maize genomes. The full-length sequence alignment of ZmPLD15 proteins (Figures S1 and Figure 4a) were highly consistent, and revealed only a few amino acid mutations. Notably, these variant amino acids were not located in functionally critical domains (e.g., the catalytic domain or substrate-binding pocket). Specifically, the two conserved HKD motifs (“HxKxxxxD”)—core elements for PLD hydrolytic activity—exhibited 100% sequence identity among all 24 ZmPLD15 proteins (Figure 4a), confirming strict conservation of the catalytic core. Consistent with the sequence conservation, the three-dimensional (3D) structures of ZmPLD15 proteins (predicted via Swiss-Model) showed no obvious conformational differences (Figure S1). Molecular docking with the substrate 1,2-diacyl-sn-glycero-3-phosphocholine (using AutoDock Vina v.1.2.7) further revealed that the substrate-binding positions of all ZmPLD15 variants were nearly identical, with minimal variations in binding free energies (ranged from −6.1 kcal/mol to −5.5 kcal/mol; Figure S1).

To evaluate the impact of geographical adaptation on substrate binding, we compared the substrate binding free energies of ZmPLD15 proteins from tropical and temperate maize germplasms (Figure 4b,c). Statistical analysis (Student’s *t*-test) showed no significant difference in binding free energy between the two groups (*p* > 0.05), indicating that the substrate binding ability of ZmPLD15 remains conserved regardless of the maize’s thermal adaptation origin.

### 2.5. Impact of ZmPLD15 Overexpression on Cold Stress Tolerance in Maize

**Figure 5:**
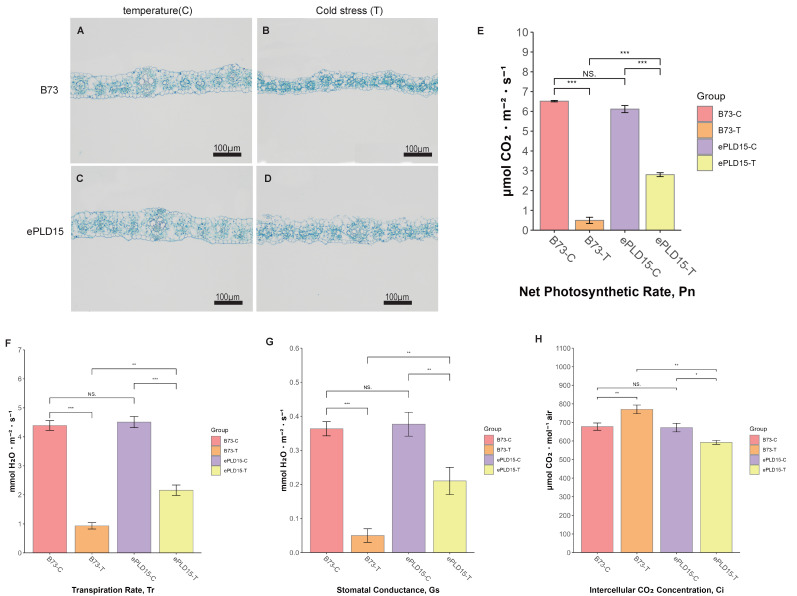
Leaf anatomical structure and photosynthetic parameters of WT (B73) and ePLD15 (*ZmPLD15*-overexpressing transgenic line, B73 background) under normal temperature and cold stress. (**A**–**D**) Leaf cross-sections (scale bar = 100 μm): (**A**) WT at normal temperature (25–28 °C, control, C); (**B**) WT after cold stress (8–10 °C, 6 h) + 24 h recovery (T); (**C**) ePLD15 at C; (**D**) ePLD15 at T. (**E**–**H**) Photosynthetic parameters: (**E**) Pn (μmol CO_2_ m^−2^ s^−1^); (**F**) Gs (mol H_2_O m^−2^ s^−1^); (**G**) Tr (mmol H_2_O m^−2^ s^−1^); (**H**) Ci (μmol mol^−1^). Data are mean ± SD (n = 3 biological replicates); statistical significance was determined by Student’s *t*-test (* *p* < 0.05, ** *p* < 0.01, *** *p* < 0.001, ns = no significant difference). C = control (continuous normal temperature); T = cold stress + 24 h recovery.

To elucidate the functional role of *ZmPLD15* in maize cold stress tolerance, we established a comparative experimental system using *ZmPLD15*-overexpressing (ePLD15) transgenic line (background: B73) and non-transgenic wild-type (WT) B73. Before phenotypic analysis, qRT-PCR was performed to verify *ZmPLD15* expression levels in WT and ePLD15 lines. Under normal temperature, the relative expression of *ZmPLD15* in ePLD15 was 17.21-fold higher than that in WT; under cold stress + 24 h recovery, the expression level in ePLD15 was 16.46-fold higher than in WT (Supplementary Table S2). These results confirm that the *CaMV 35S* promoter effectively drove stable overexpression of *ZmPLD15* in the transgenic lines, providing a reliable genetic background for subsequent cold tolerance assays. Both genotypes were subjected to cold stress treatment, and the structural integrity of leaf cells—an important indicator of cold-induced cellular damage—was observed via microscopic sectioning. As shown in Figure 5A–D, under normal temperature conditions, the leaf cells of both WT and ePLD15 plants exhibited a regular, intact structure with clear cell boundaries and full cytoplasm. However, after cold stress exposure, significant differences in cellular morphology emerged between the two genotypes: WT B73 leaves showed obvious signs of cell dehydration and wilting, including shrunken cytoplasm, disrupted cell membranes, and disorganized mesophyll cell arrangement—typical symptoms of cold-induced cellular damage. In contrast, although the leaf cells of ePLD15 plants also showed mild damage (e.g., slight cytoplasmic shrinkage), they maintained significantly better cell integrity compared to WT plants, with more intact cell membranes and a relatively organized mesophyll structure. This observation directly indicates that overexpression of *ZmPLD15* enhances the structural stability of maize leaf cells under cold stress, thereby reducing cold-induced cellular damage.

To further evaluate the protective effect of *ZmPLD15* on maize physiological function under cold stress, we measured key photosynthetic parameters of two materials—non-transgenic maize (WT, B73) and *ZmPLD15*-overexpressing transgenic line (ePLD15, B73 background)—one day after cold stress recovery, including Pn, Gs, Tr, and Ci. The full process was: three-leaf-stage seedlings were treated with cold stress (8–10 °C, 6 h) and then recovered at 25–28 °C for 24 h before measurement. These parameters are critical for assessing the photosynthetic capacity and stress recovery ability of plants. The results showed that the photosynthetic function of ePLD15 plants recovered more effectively compared to WT plants. Specifically, the Pn, Gs, and Tr of ePLD15 plants were maintained at approximately 45.8%, 47.9%, and 55.8% of their pre-stress levels, respectively, while the Ci was around 88.1% of the pre-stress value—indicating that the photosynthetic apparatus of ePLD15 plants retained a relatively high level of activity after cold stress. In sharp contrast, the photosynthetic parameters of WT plants were severely impaired: Pn, Gs, and Tr dropped to only 7.6%, 21.3%, and 13.8% of their pre-stress levels, respectively. Notably, the Ci of WT plants increased to 113.7% of the pre-stress value, which is likely due to the severe inhibition of photosynthetic CO_2_ fixation (resulting from damaged photosynthetic machinery) while stomatal conductance remained partially open, leading to the accumulation of CO_2_ in the intercellular space. Statistical analysis confirmed that all the observed differences in photosynthetic parameters between ePLD15 and WT plants were significant (*p* < 0.05). These results collectively demonstrate that overexpression of ZmPLD15 not only protects the structural integrity of leaf cells but also effectively maintains the photosynthetic function of maize under cold stress, thereby enhancing the cold tolerance and stress recovery ability of maize plants.

## 3. Discussion

Pangenome-based gene family analysis effectively captures genetic variation overlooked by single reference genomes [10,42]. In this study, we identified 21 *PLD* family members from the maize pangenome constructed with 26 genomes, among which five were classified as private genes—present in only one of the 26 genomes. In contrast, the most widely used B73 reference genome contains only 15 *PLD* genes, and of the 21 pan-genes, merely nine are core genes (present in all 26 genomes with allelic variants in each). This highlights the pangenome’s advantage in complete gene family identification. Notably, *ZmPLD14* and *ZmPLD17* exhibit a “complementary distribution” across genomes (presence of one correlates with absence of the other), implying functional redundancy to maintain PLD-mediated pathway stability [10,43].

The calculation of Ka/Ks for homologous *PLD* genes across different genomes provides critical insights into the evolutionary forces shaping the gene family during maize domestication and breeding [44]. Ka/Ks analysis of homologous *ZmPLD* genes revealed most *ZmPLD* genes under purifying selection (Ka/Ks < 1), reflecting their conserved roles in maize growth (e.g., membrane homeostasis, signal transduction) [45,46]. Notably, only three *PLD* genes (including *ZmPLD15*) showed Ka/Ks ratios > 1. Among these, *PLD15* had a distinct Ka/Ks distribution peak shifted toward Ka/Ks > 1, indicating stronger positive selection—suggesting its variants confer adaptive advantages (e.g., enhanced cold tolerance) [47].

SVs in regulatory regions (especially promoters) drive transcriptional plasticity, supporting plant environmental adaptation and response to breeding selection [48]. SVs in the *ZmPLD15* promoter regulate its transcription. Relative to B73, five shared SVs were identified (2 deletions, 3 insertions). Region1 (with ERF/bHLH binding sites) and Region5 (with GATA and G2-like binding sites) significantly affected *ZmPLD15* expression (varieties carrying Region1 deletion or Region5 insertion showed distinct expression levels), while Regions2–4 (no functional TF binding sites) had no impact—confirming only SVs with functional elements mediate transcriptional regulation [49,50,51,52,53]. “GCATGTGC”, “GCACATGC” and “CCTCTGCCTTCTTCATGGCCA” are located in Region1 SV of ZmPLD15. As typical TF binding sites MP00084 [54,55,56,57], MP00101 [58,59,60] and MP00456 [61,62,63,64], they have been repeatedly experimentally verified in model plants such as *Arabidopsis thaliana* that their downstream gene expression is regulated by ERF/bHLH—type TFs. And “GAGATTCTGA” in Region5 SV of ZmPLD15 serves as the recognition site MP00022 [65] for G2-like TFs, and “TTGTCATCAGCAACA” serves as the recognition site MP00369 [66] for GATA—type TFs, which have also been experimentally verified in *Arabidopsis thaliana*. In contrast to the variable promoter, ZmPLD15′s coding region is highly conserved. ZmPLD15 proteins from different genomes showed minimal differences in secondary/tertiary structures (including catalytic HKD motifs and substrate-binding pockets) [67]. Molecular docking revealed 1,2-diacyl-sn-glycero-3-phosphocholine binding free energies ranged from −6.1 to −5.5 kcal/mol, with no significant differences between tropical and temperate germplasms (*p* > 0.05). This indicates strict evolutionary constraint on *ZmPLD15*′s catalytic function to ensure stable physiological roles [68].

This “variable promoter-conserved coding region” pattern reflects breeding preferences for *ZmPLD15*: regulatory SVs fine-tune expression (e.g., cold-induced *ZmPLD15* upregulation) to enhance adaptability, while coding-region mutations (which risk disrupting protein function, e.g., membrane metabolism disorders) are avoided—aligning with the general trend of “regulatory variation driving phenotypic optimization” in crop improvement [69,70,71,72,73].

The cold tolerance advantage conferred by *PLD15* overexpression first manifests in the protection of mesophyll cell photosynthetic activity—a process highly sensitive to cold stress [74,75]. Low temperatures typically disrupt the structure of thylakoid membranes (the site of light-dependent reactions) and induce the inactivation of Rubisco (the key enzyme in Calvin cycle carbon fixation), leading to non-stomatal limitations of photosynthesis [76]. Functional validation showed ePLD15 had better leaf cell integrity than WT after cold stress (8–10 °C, 6 h): ePLD15 exhibited less cytoplasmic shrinkage and membrane damage. Photosynthetic recovery was also superior in ePLD15 retained 45.8% Pn, 47.9% Gs, and 55.8% Tr of pre-stress levels, with Ci at 88.1%. In contrast, WT retained only 7.6% Pn, 21.3% Gs, and 13.8% Tr, with Ci elevated to 113.7% (CO_2_ accumulation from non-stomatal limitations). Together, these confirm *ZmPLD15* enhances maize cold tolerance by protecting the photosynthetic system, providing a key gene for cold-tolerant breeding. Our study focuses on cold stress, but ZmPLD genes may have broader functions in other abiotic stresses. As reported, *ZmbZIP54* is involved in maize Pb tolerance [77], and salt stress affects maize seed germination via specific genetic regulation [78], suggesting *ZmPLD* genes might be part of these multi-stress response networks.

## 4. Materials and Methods

### 4.1. Identification of Maize PLD Gene Family

Twenty-six maize genomes were obtained from the study by Hufford et al. [41]. For *PLD* gene identification, the HMM profile of the PLD domain (PF00614) was downloaded from the Pfam database (https://pfam.xfam.org/). HMMER 3.3.2 was used to search the annotated genes of the 26 genomes; genes with E-value < 10^−5^ and PLD domain alignment length > 70% were designated as ZmPLD members.

### 4.2. Phylogenetic Analysis and Presence/Absence Variation in ZmPLD Gene Family

For PLD phylogenetic analysis, protein sequences from maize, Arabidopsis, and rice were used: MAFFT v.7.526 for multiple alignment, Trimal 1.5.rev for trimming conserved regions, IQTree v.3.0.1 for tree building, and R’s ggtree v.3.8.2 for visualization. *ZmPLD* PAV data were from Hufford et al. [41]; gene IDs and presence/absence in 26 maize genomes were extracted, then visualized via Rscript v.4.3.3 with the ComplexHeatmap package v.2.13.1.

### 4.3. Ka/Ks Calculation

Protein and full-length coding sequence (CDS) sequences of identified *ZmPLD* genes were extracted from different maize genomes. Ka/Ks values were calculated using the KaKs Calculator v.3.0 with the ‘YN’ model [37,79], and density plots of these values were generated with R’s ggridges package v.0.5.6. Additionally, the proportion of *ZmPLD* genes with Ka/Ks > 1 was computed, and a heatmap of this proportion was constructed using R’s pheatmap package v.1.0.12.

### 4.4. Expression and Structure of ZmPLD15 Genes Are Affected by SV in the Promoters

For *ZmPLD15*, 2000-bp upstream sequences were extracted from maize genomes containing this gene. MAFFT v.7.526 was used for multiple sequence alignment, with B73 as the reference. Python v.3.10.13 was employed to identify insertions/deletions (≥50 bp) present in at least 5 promoter sequences (defined as SVs). SV sequences were extracted, and their TF binding sites were predicted via FIMO. Samples were grouped by the presence/absence of each SV, and the significance of SVs’ association with ZmPLD15 expression was analyzed. Finally, Python’s matplotlib library was used for visualization of the results.

### 4.5. Impact of Mutations on ZmPLD15 Protein Spatial Structure and Substrate Binding Free Energy

The sequence conservation and the 3D structures of ZmPLD15 proteins encoded by different maize genomes were conducted by MAFFT and predicted via the SwissModel website. Molecular docking of these proteins with the substrate (1,2-diacyl-sn-glycero-3-phosphocholine) was performed using AutoDock Vina v.1.2.7 to calculate binding free energies, and 3D visualization of docking results was done with PyMOL v.2.5.2. Based on phylogenetic trees and breeding information, representative temperate and tropical maize germplasms were selected. Statistical analysis of ZmPLD15 expression differences between the two groups was conducted, and results were visualized using Python’s matplotlib library.

### 4.6. Impact of ZmPLD15 Overexpression on Cold Stress Tolerance in Maize

The ZmPLD15 overexpression vector was constructed using the commercial vector pCAMBIA3301 (CAMBIA, Canberra, Australia). The CDS of ZmPLD15 (derived from the B73 genome, gene ID: *Zm00001eb103990*) was cloned and inserted into the plant expression vector pCAMBIA3301 (replacing the *GUS* gene) via digestion-ligation using *Nco*I and *BstE*II sites. Agrobacterium-mediated genetic transformation and glufosinate screening yielded ZmPLD15-overexpressing transgenic B73 maize mutants. Three-leaf-stage seedlings of WT and ePLD15 were subjected to cold stress (8–10 °C for 6 h), followed by 24 h recovery at room temperature. Quantitative real-time PCR (qRT-PCR) with three biological replicates of WT and ePLD15 maize seedlings were performed, and Specific primers for *ZmPLD15* and *ZmGAPDH* are listed in Supplementary Table S3. Leaf section preparation followed the protocol described by Atkinson [80] with minor modifications for maize: 1 cm mid-leaf segments (avoid midrib) fixed in FAA (1:1:18, *v*/*v*, 4 °C, 24 h), dehydrated (gradient ethanol), embedded in paraffin (52–56 °C), sectioned (8 μm), stained (1% safranin O, 0.5% fast green), and imaged with Olympus BX53(Olympus Corporation, Hachioji, Tokyo, Japan), 400×. A CIRAS-2 portable photosynthesis system measured photosynthetic parameters (Pn, Gs, Tr, Ci) of WT and transgenic lines under room temperature and cold stress. Data were statistically analyzed and visualized using R’s ggplot2 package 3.5.1.

## Data Availability

No new raw data were generated in this study. All data analyzed during the research were obtained from the publicly available pan-maize genome dataset reported by Hufford et al. (2021) [41]. The processed data generated during this study, including multiple sequence alignment results, Ka/Ks ratio calculation outputs, and phylogenetic tree annotation files, are available from the corresponding author (Yunpeng Wang, wangypbio@163.com) upon reasonable request.

## Supplementary Materials

The following supporting information can be downloaded at https://www.mdpi.com/article/10.3390/plants14243858/s1, Figure S1: Spatial structure simulation of ZmPLD15 proteins derived from different maize genomes and their molecular docking results with the substrate (1,2-diacyl-sn-glycero-3-phosphocholine); Table S1: Pan_gene list. Table S2: qRT-PCR Results for ZmPLD15 Overexpression Validation. Table S3: Primers Used for qRT-PCR.
