# Supplementary figures and images for: Pangenome-Wide Identification, Evolutionary Analysis of Maize ZmPLD Gene Family, and Functional Validation of ZmPLD15 in Cold Stress Tolerance
Source: Plants (Basel). 2025 Dec 18;14(24):3858. doi: 10.3390/plants14243858 (PMC12737115; doi:10.3390/plants14243858)

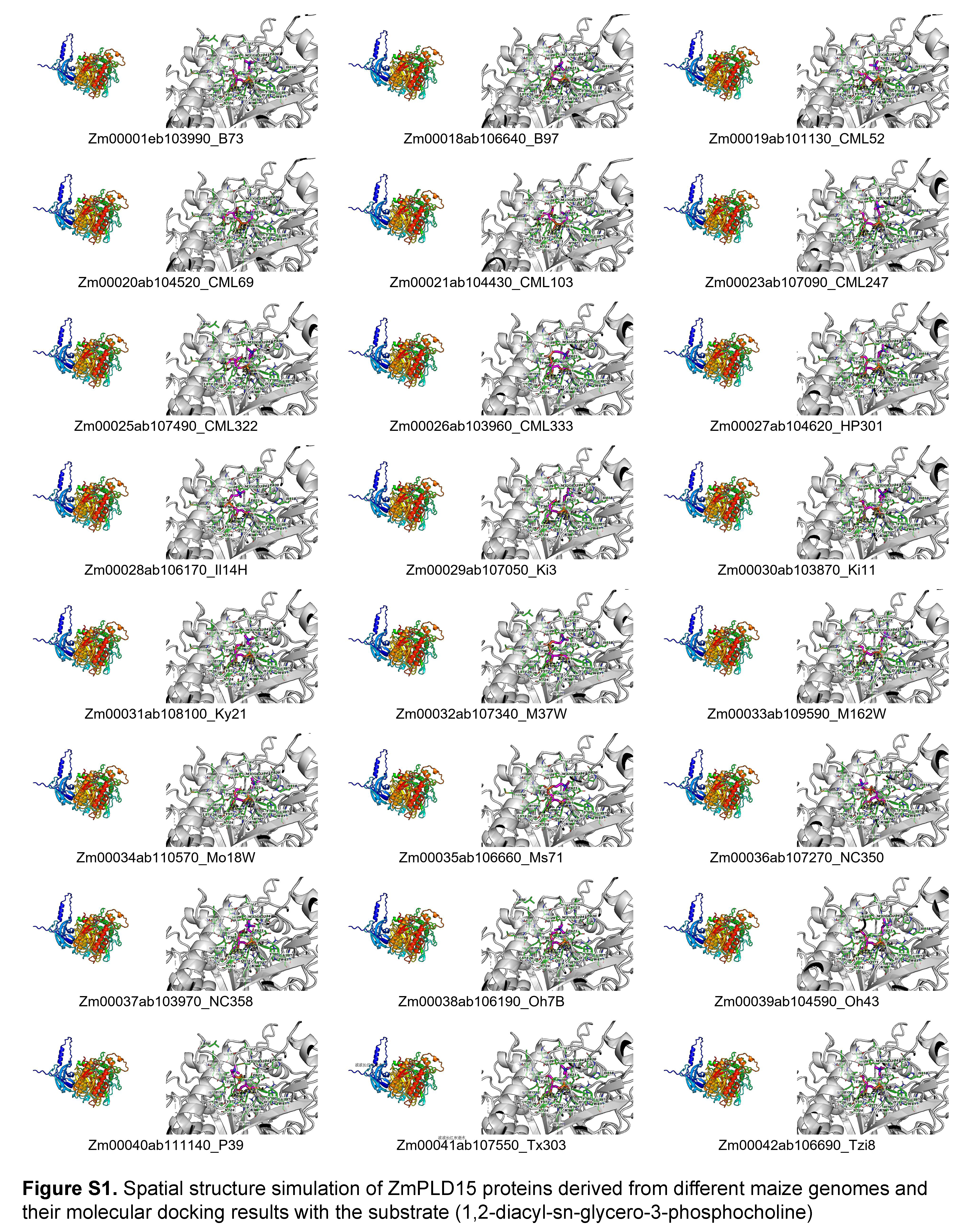

Supplement: Supplementary file 1 [file plants-14-03858-s001.zip › FigS1.tiff]
